# NEK6 functions as an oncogene to promote the proliferation and metastasis of ovarian cancer

**DOI:** 10.7150/jca.103769

**Published:** 2025-01-13

**Authors:** Sainan Gao, Min Su, Tingting Bian, Yifei Liu, Yanhua Xu, Yuquan Zhang

**Affiliations:** 1 Suzhou Medical College of Soochow University, Suzhou 215123, China.; 2 Department of Obstetrics and Gynecology, Affiliated Hospital of Nantong University, Nantong 226001, China.; 3 Department of Pathology, Affiliated Hospital of Nantong University, Nantong 226001, China.

**Keywords:** NEK6, ovarian cancer, prognosis, metastasis, proliferation

## Abstract

**Background:** Ovarian cancer (OC) is a common malignant tumor of the female reproductive organs. The novel serine/threonine kinase NEK6 is highly expressed in various cancers and affects the prognosis of patients. However, the role of NEK6 in OC is still unclear.

**Methods:** In this study, the expression profiles of NEK6 in OC and its roles in the development of OC were investigated. The expression profiles of NEK6 across cancers and OC were explored using bioinformatics analysis, and its expression in OC patients was detected by immunohistochemical (IHC) staining. The correlation between its expression and clinicopathological factors was also analyzed. Furthermore, the NEK6 levels in the tumor tissues of OC patients were detected via RT‒qPCR and Western blotting. Biological functions, including cell growth, migration, invasion and apoptosis, were analyzed using MTT, Transwell and flow cytometry assays, respectively.

**Results:** Bioinformatics analysis revealed that NEK6 was highly expressed in most human cancers, including OC. IHC revealed 67.27% moderate or strong NEK6 staining in tumor tissues, 32.73% (36/110) weak staining, and negative or weak NEK6 staining in normal ovarian tissues, and its high expression was correlated with clinicopathological factors, including histological grade (*P*=0.008) and metastasis (*P*=0.006). The Kaplan‒Meier survival curve revealed that OC patients with high expression of NEK6 had poorer overall survival rates (*P*=0.025). NEK6 was overexpressed in OC tissues and SK-OV-3 and A2780 cells, and when NEK6 was knocked down with siRNAs, cell growth, migration and invasion were inhibited, whereas cell apoptosis was significantly promoted.

**Conclusion:** NEK6 is highly expressed in OC; its overexpression indicates poor prognosis; and NEK6 knockdown leads to inhibited growth, migration and invasion while promoting the apoptosis of OC cells. These findings indicate that NEK6 is a potential oncogene and a poor prognostic factor in OC, suggesting that NEK6 can serve as a new therapeutic candidate for OC and that NEK6 inhibition may be an effective strategy for OC treatment.

## Introduction

Ovarian cancer (OC) is one of the most common malignant tumors in women worldwide, and its incidence rate is the third highest after that of only cervical carcinoma and endometrial carcinoma 1. Malignant ovarian cancer, especially epithelial cancer, is difficult to detect early and has unknown causes. Apart from hereditary OC, primary prevention measures are not available. Currently, early diagnosis and treatment are advocated for early detection of lesions. Therefore, discovering biomarkers for the early diagnosis of OC and finding new molecular therapeutic targets are the primary tasks in conquering OC and are currently hot topics in the study of the molecular pathological mechanisms of OC 2.

The never in mitosis gene A (NIMA)-related kinase (NEK) family was originally found in fungi and consists of 11 serine/threonine kinase members, including NEK1-11, which are considered key initiators of mitosis 3. NEKs have been identified in various important cellular processes. Unlike those of traditional kinase families, the kinase characteristics of NEKs are not distinct, and their role in cell cycle regulation was initially known. Recent studies have shown that NEKs play roles in cell cycle arrest 4, the DNA damage response, mRNA splicing, mitochondrial homeostasis, genome organization and primary cilia function in mammals 5. Recently, NEKs were shown to be abnormally expressed in different cancers and play vital roles in the development of cancer 6. However, the molecular mechanism of NEKs in tumorigenesis is still unclear.

NEK6 is a serine/threonine kinase and a new member of the NEK family 7, and it has been reported that NEK6 is essential for promoting cell cycle progression by participating in cell mitosis 8. Inactivation of NEK6 leads to cell apoptosis and mitotic arrest. In addition, NEK6 is necessary for cell cycle progression. Recent studies have also revealed that NEK6 is a new checkpoint of DNA damage, and its inactivation is crucial for G2/M phase arrest after DNA damage during the cell cycle 9. Studies have also shown that NEK6 plays potential roles in several cancers 10, including prostate cancer (PC) 5, head and neck squamous cell carcinoma (HNSCC) 11, hepatocellular carcinoma (HCC) 12, and breast cancer (BC) 13. In addition, it has been reported that NEK6 is associated with hypoxia in OC 14, but its mechanism has not been explored. In this study, the expression of NEK6 in OC and its role in the progression of OC were explored.

## Methods

### Data collection and bioinformatics analysis

The data of OC patients and normal controls from the databases of the Tumor IMmune Estimation Resource (TIMER2.0, https://cistrome.shinyapps.io/timer/) 15; differential gene expression analysis in tumor, normal, and metastatic tissues (TNMplot, https://tnmplot.com/analysis2/) 16; and the University of ALabama at the Birmingham CANcer data analysis portal (UALCAN, https://ualcan.path.uab.edu/index.html) 17 were used for pancancer analysis of NEK6 expression. Furthermore, the expression of NEK6 in OC patients was analyzed using the data from the TCGA dataset (https://portal.gdc.com) and GEPIA2 (http://gepia2.cancer-pku.cn/) 18, and normal tissue data were obtained from Genotype-Tissue Expression (GTEx) cohorts 19.

### Human species

Human OC clinical tumor tissues were obtained for observation of NEK6 expression in patients with OC. In total, 112 OC tumor tissues were paraffin-embedded and sectioned for NEK6 immunohistochemical staining. Thirty fresh OC tissues and paired normal ovarian tissues were collected to detect NEK6 expression via RT‒qPCR and Western blot methods. All clinical tissues were collected from OC patients at the Department of Obstetrics and Gynecology, Affiliated Hospital of Nantong University, from January 2005 to December 2010. Written informed consent was obtained from each participant, and the study was approved by the Ethics Committee of the Affiliated Hospital of Nantong University (Approval no. 2023-L002).

### Immunohistochemical (IHC) staining

The tissue microarray sections containing paraffin-embedded OC tumor tissue samples were prepared by the Department of Pathology, Affiliated Hospital of Nantong University (Nantong, China). IHC staining was performed using the Envision Plus/Horseradish Peroxidase Kit (DAKO, USA). In brief, the sections were incubated with a NEK6 antibody (1:50 dilution) (Proteintech, #10378-1-AP) overnight at 4 °C. After washing in PBS, the sections were incubated with the Envision Plus secondary antibody at 37 °C for 30 min, with diaminobenzidine solution for 5 min and then with hematoxylin solution.

The staining intensity of NEK6 in OC tumor tissues was independently evaluated by two pathologists and scored as 0 (negative), 1 (weak), 2 (moderate) or 3 (strong). The score based on the percentage of positive cells was 0 (≤5%), 1 (6-25%), 2 (26-50%), 3 (51-75%) or 4 (>75%). The scoring results were assessed on the basis of the intensity and percentage scores. NEK6 expression is presented as “-” (negative, score of 0), “+” (weak, score of 1-4), “++” (moderate, score of 5-8), and “+++” (strong, score of 9-12).

### Cells, culture and treatments

The human OC cell lines SK-OV-3, A2780, and IOSE-80 (a normal human ovarian epithelial cell line) were used as normal controls, and all cells were obtained from the Shanghai Institute of Biochemistry and Cell Biology (SIBCB) (China). SK-OV-3 cells were cultured in RPMI 1640 medium (Thermo Fisher Scientific, USA) supplemented with 10% fetal bovine serum (FBS) (Thermo Fisher Scientific, USA), and A2780 and IOSE-80 cells were cultured in Dulbecco's modified Eagle's medium (DMEM) (Thermo Fisher Scientific, USA) supplemented with 10% FBS (Thermo Fisher Scientific, USA). All the cells were cultured in an incubator at 37 °C with 5% CO_2_.

NEK6-specific siRNAs (si-NEK6_1, si-NEK6_2 and si-NEK6_3) were predesigned and screened for NEK6 inhibition in OC cells, and a negative control siRNA (si-NC) was designed according to the nonhuman homologous siRNA sequence. According to the manufacturer's protocols, siRNA transfection was performed using Lipofectamine^®^ 2000 (Thermo Fisher Scientific, USA). All siRNAs used here were obtained from Biomics Biotechnologies Co., Ltd. (China), and the sequences are shown in Table 1. si-NEK6_2 (si-NEK6) was selected for functional experiments.

### Real-time quantitative PCR (RT‒qPCR)

The mRNA levels of genes expressed in OC tissues or cells were detected via RT‒qPCR. Briefly, the OC tissues used for RT‒qPCR detection were freshly frozen, and approximately 50 mg of each tissue sample was subjected to total RNA extraction. At the cellular level, SK-OV-3 and A2780 or IOSE-80 cells in the logarithmic growth phase were inoculated into 24-well plates at a density of 5×10^4^ cells per well. After culturing for 24 h at 37 °C with 5% CO_2_, the cells were subjected to siRNA transfection according to the above method. Total RNA was extracted from tissues or cells via TRIzol^®^ reagent (Thermo Fisher Scientific, USA) according to the manufacturer's protocol. GAPDH was used as an internal control. First-strand cDNA was synthesized via a RevertAid First Strand cDNA Synthesis Kit (Thermo Fisher Scientific, #K1622), and the qPCRs were carried out using DyNAmo Flash SYBR-Green qPCR (Thermo Fisher Scientific, #F415XL) according to the manufacturer's protocol. The results were analyzed using the 2^-ΔΔCt^ method 20.

The sequences of primers used were as follows: NEK6 forward, 5'-TCTTGAAGCAACTGAACCA-3', and reverse, 5'-CCAACTCCAGCACAATGT-3'; GAPDH forward, 5'-GGAGCGAGATCCCTCCAAAAT-3', and reverse, 5'-GGCTGTTGTCATACTTCTCATGG-3'.

### Western blot

The protein levels of genes expressed in OC tissues and cells were detected using Western blot. Briefly, the OC tissues used for protein level detection were freshly frozen, and approximately 100 mg of each tissue sample was subjected to total protein extraction. At the cellular level, SK-OV-3 and A2780 or IOSE-80 cells in the logarithmic growth phase were plated onto 6-well plates at a density of 1×10^5^ cells per well. After culturing for 24 h at 37 °C with 5% CO_2_, the cells were subjected to siRNA transfection according to the above method. Total protein was extracted from the tissues or cells via RIPA lysis buffer (Beyotime) according to the manufacturer's protocols. Total protein was quantified with a BCA kit (Beyotime), separated by sodium dodecyl sulfate (SDS)-polyacrylamide gel electrophoresis (PAGE), and then transferred onto PVDF membranes (MerckMillipore). The membranes were subsequently subjected to antigen blockade using 5% skim milk at 37 °C for 2 h and then incubated with an anti-NEK6 antibody (1:500 dilution) (Proteintech, #10378-1-AP) or an anti-GAPDH antibody (1:5,000 dilution) (Proteintech, #60004-1-Ig) at 4 °C overnight. After washing three times in TBST for 1 min, the membranes were incubated with horseradish peroxidase (HRP)-conjugated IgG (1:5,000 dilution) at 37 °C for 1 h. After washing in TBST for 5 min three times, the protein bands were detected using BeyoECL Plus (Beyotime), and the blots were quantified using ImageJ software.

### MTT assay

The viability of OC cells was detected via the MTT assay. In brief, SK-OV-3 and A2780 cells in the logarithmic growth phase were adjusted to 1×10^5^ cells/mL and then inoculated into 96-well plates with 100 μL of cells per well. After culturing for 24 h at 37 °C with 5% CO_2_, the cells were subjected to siRNA transfection according to the above method. After culturing in a 5% CO_2_ incubator at 37 °C for 4‒6 h, the cell supernatant was removed, and the cells were washed twice with serum-free medium. Then, 100 μL of DMEM supplemented with 10% FBS was added. After culturing for 48 h, 10 μL of MTT solution per well was added, and the mixture was incubated at 37 °C in the dark for 3-4 h. The culture medium was replaced with 150 μL of DMSO per well, and the mixture was incubated for 10 min at room temperature. The OD values at the same time points were measured via a microplate reader (Thermo Fisher Scientific, MK3) at a wavelength of 492 nm, and the measured OD values were used for cell viability analysis.

### Wound-healing assay

The migration abilities of OC cells were detected via a wound-healing assay. Briefly, SK-OV-3 and A2780 cells in the logarithmic growth phase were plated onto 96-well plates at a density of 1×10^5^ cells per well. After culturing for 24 h at 37 °C with 5% CO_2_, the cells were subjected to siRNA transfection according to the above method. After 48 h of transfection, the cells were inoculated into 3.5 cm culture dishes with 1×10^5^ cells per dish. After culturing at 5% CO_2_ and 37 °C for 24 h, the wounds were created via a 200 μL pipette tip through confluent monolayer cells. The floating cells were subsequently washed with PBS, and the cells were subsequently cultured at 37 °C with 5% CO_2_ for 48 h. Finally, the migrating cells were observed and photographed under a microscope, and the migration distance of the cells was calculated using ImageJ software.

### Transwell assay

The migration abilities of OC cells were detected by a Transwell assay. In brief, SK-OV-3 and A2780 cells in the logarithmic growth phase were collected and adjusted to 5×10^5^ cells/mL. Transwells were placed on a 24-well plate, 200 μL of cell suspension was added to the upper chamber, and 600 μL of medium was added to the lower chamber of each transwell. The cells were subjected to siRNA transfection according to the above method and cultured for 72 h at 37 °C with 5% CO_2_. After the chamber was removed from the plates, the medium of the upper chamber was discarded, and the samples were carefully wiped with a cotton swab. The cells in the chamber were washed with preheated PBS twice, fixed with 4% precooled paraformaldehyde for 30 min, and stained with crystal violet for 10 min. The polycarbonate membrane in the upper chamber was carefully removed and observed under a microscope. The cell numbers were calculated by ImageJ software.

### Matrigel-based transwell assay

The invasive abilities of OC cells were detected by a Matrigel-based transwell assay. Briefly, Matrigel (BD Biosciences) was thawed at 4 °C overnight and diluted with serum-free DMEM at a 3:1 ratio. After the transwells were placed on a 24-well culture plate, 100 μL of Matrigel was added to the upper chamber, and the mixture was incubated for 2-6 h at 37 °C. SK-OV-3 and A2780 cells in the logarithmic growth phase were collected and adjusted to 5×10^5^ cells/mL in DMEM without FBS, 300 μL of each cell suspension was added to the upper chamber, and 600 μL of medium was added to the lower chamber. The cells were subjected to siRNA transfection according to the above method and cultured for an additional 72 h. After the upper chamber was removed from the plate, the medium of the upper chamber was discarded, and the noninvasive cells were wiped carefully with a cotton swab. The cells in the chamber were washed with preheated PBS twice, fixed with 4% precooled paraformaldehyde for 30 min, and stained with crystal violet for 10 min. The polycarbonate membrane in the upper chamber was carefully removed and observed under a microscope. The number of cells was calculated using ImageJ software.

### Flow cytometry

OC cell apoptosis was detected by flow cytometry. In brief, SK-OV-3 and A2780 cells in the logarithmic growth phase were collected and plated onto 6-well plates at a density of 3×10^5^ cells/well. After culturing for 24 h at 37 °C with 5% CO_2_, the cells were subjected to siRNA transfection according to the above method and cultured for 48 h. After cell collection was performed by centrifugation at 1,500 rpm at room temperature for 5 min, the cells were resuspended once with precooled PBS and then centrifuged at 1,500 rpm for 5 min. After resuspending in 300 µL of 1×binding buffer, 5 µL of Annexin V-FITC (Beyotime) was added, and the mixture was incubated for 15 min in the dark at room temperature, followed by 10 µL of propidium iodide (PI) (Beyotime) staining for 10 min at room temperature in the dark. Finally, apoptotic cells were detected with a flow cytometer (BD Biosciences), and the results were analyzed using FlowJo 7.6 software.

### Statistical analysis

All data in the study are presented as the mean value ± standard deviation (SD). SPSS 20.0 and GraphPad Prism 8.0 software were used to carry out the statistical analysis. Comparisons between two groups were performed using Student's *t* test. Comparisons among three or more groups were performed using one-way analysis of variance (ANOVA) followed by Dunnett's *post hoc* test. The correlation between NEK6 expression and clinicopathological factors in OC patients was determined via the *Pearson x^2^* test. Survival analysis was performed via the Kaplan‒Meier method with the log-rank test. A *P* value < 0.05 was considered statistically significant.

## Results

### Overexpression of NEK6 in various human cancers and OC

NEK6 has been reported to be highly expressed in various human cancers. To further explore the expression of NEK6 in human cancers, online databases were used for analysis.

**Figure 1:**
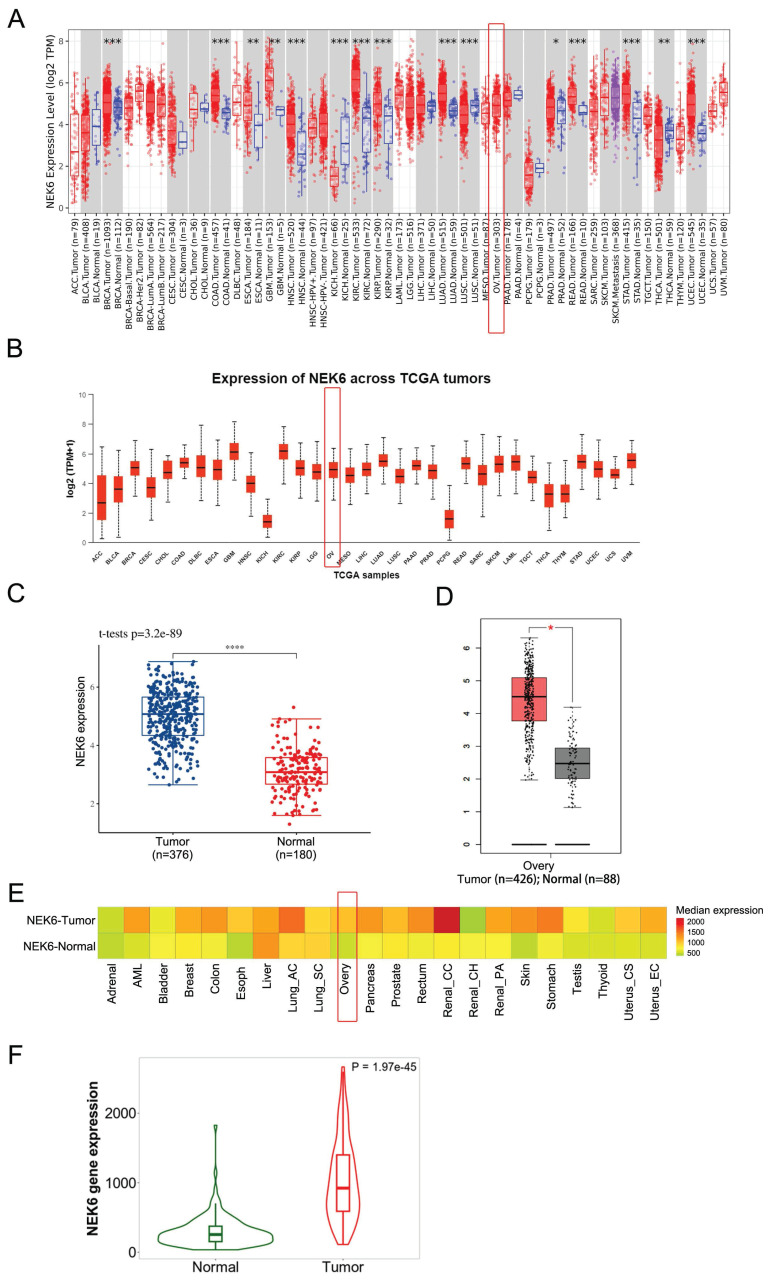
Bioinformatics analysis of NEK6 expression in human cancers and OC. **A.** Pancancer analysis of NEK6 in different human cancers via the TIMER 2.0 online dataset, OV tumors (n=303). *P<0.05; **P<0.01; ***P<0.001.** B.** Pancancer analysis of NEK6 in different human cancers via the UALCAN online dataset, OV tumors (n=305). **C.** NEK6 expression in OC tissues and normal tissues from the TCGA dataset; tumor (n=376), normal (n=180), ****P<0.001. **D.** NEK6 expression in OC tissues and normal tissues from the GEPIA2 dataset; tumor (n=376), normal (n=180), *P<0.05. **E.** Pancancer heatmap analysis of NEK6 from the TNM plot dataset. **F.** Violin plot of NEK6 expression obtained from the gene signature analysis via TNM plot, normal (n=133), and tumor (n=374) samples.

Pancancer analysis via the online TIMER2.0 and UALCAN datasets revealed that NEK6 was highly expressed in most human cancers and that NEK6 was highly expressed in OC tumors (Fig. 1A).

The data from TCGA revealed that NEK6 expression was significantly higher in OC tissues (n=376) than in ovarian tissues (n=180) (*P*<0.001) (Fig. 1C). GEPIA2 also revealed that NEK6 expression in 376 OC tumors was greater than that in 180 normal tissues (*P*<0.05) (Fig. 1D). Pancancer heatmap analysis of the TNM plot dataset also revealed its high expression in tumor tissues of the ovary (Fig. 1E). Violin plot analysis of the TNM plots revealed that NEK6 expression was higher in tumors (n=374) than in normal tissues (n=133) (*P*<0.05) (Fig. 1F).

These results indicate that NEK6 is highly expressed in most human cancers, including OC, and its expression is clearly higher in OC tumors than in normal ovaries.

### High NEK6 expression in OC patients and its correlation with clinicopathological factors

**Figure 2:**
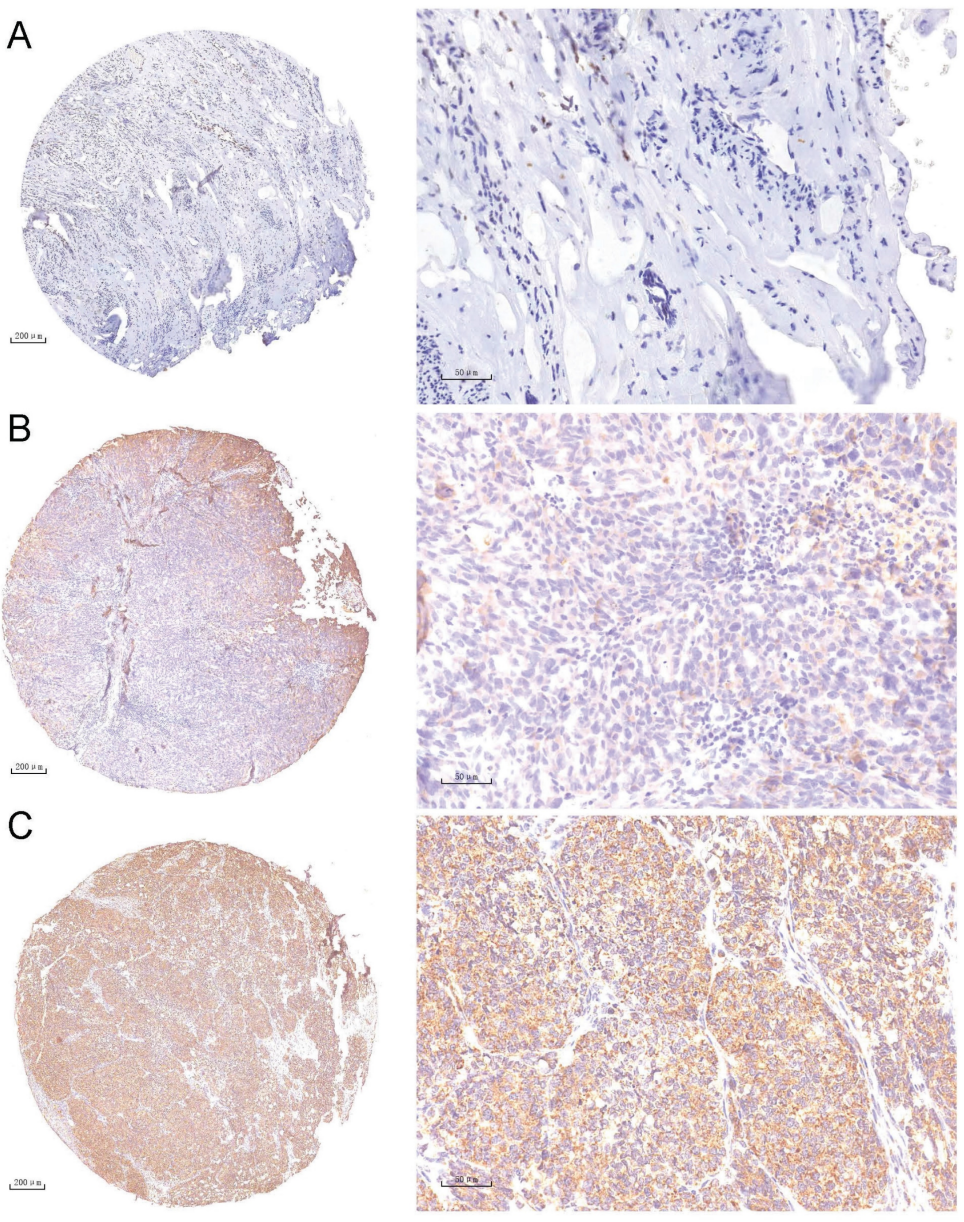
IHC staining analysis of NEK6 expression in tumor tissues from OC patients. **A.** Negative staining of NEK6 in normal ovarian tissues (A, 5×; A1, 40×); **B.** Weak staining of NEK6 in OC tissues (B, 5×; B1, 40×); **C.** Strong staining of NEK6 in OC tissues (C, 5×; C1, 40×).

A tissue chip containing 110 OC tumor tissue samples was prepared and used for NEK6 expression analysis. IHC staining revealed 67.27% (74/110) moderate and/or strong NEK6 staining in tumor tissues, 32.73% (36/110) weak staining (Table 2 & Fig. 2), and negative or weak NEK6 staining in normal ovaries.

**Figure 3:**
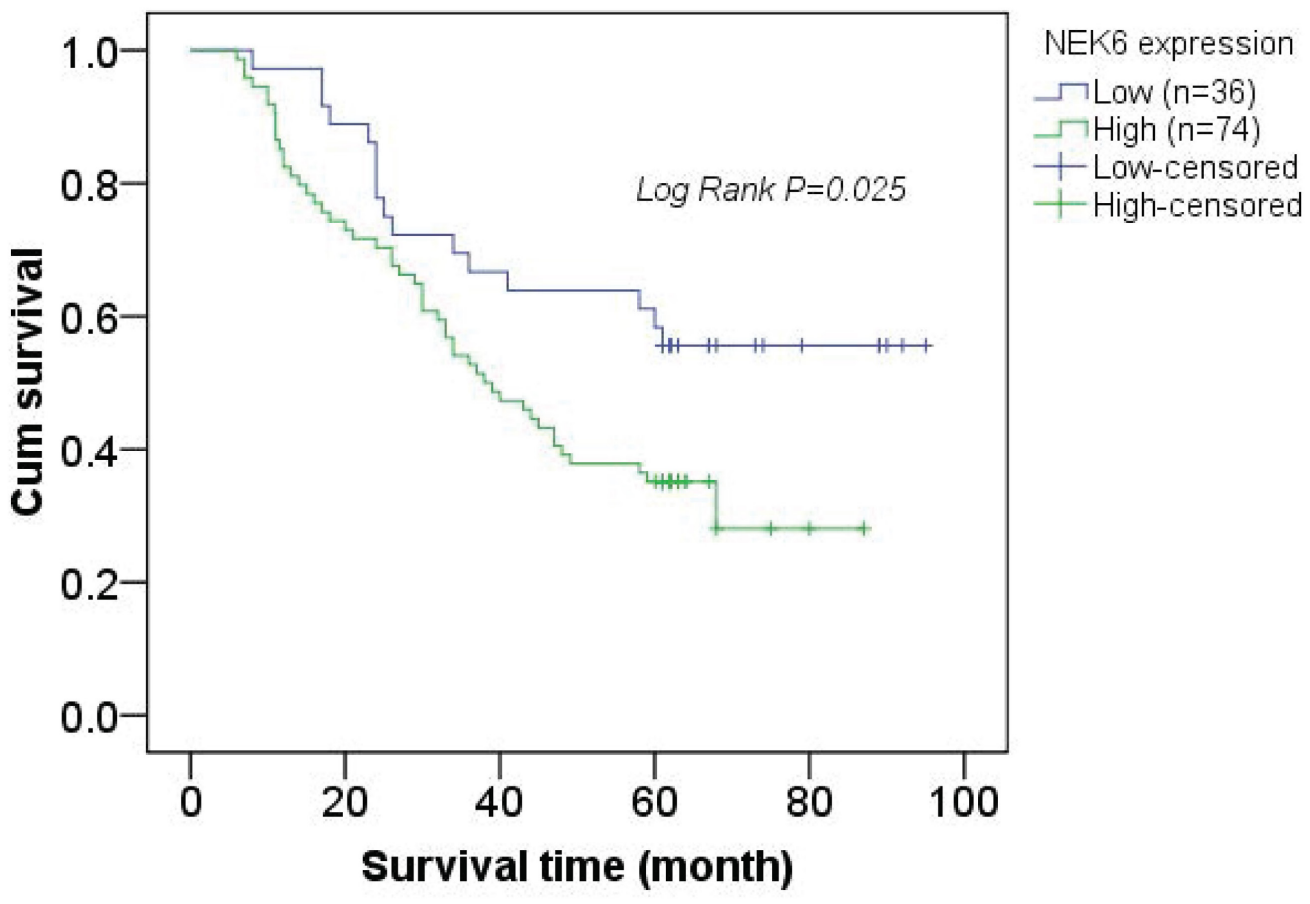
Kaplan-Meier survival curve analysis of the correlation between NEK6 expression and the survival time of OC patients. The overall survival time of OC patients with high NEK6 expression (green line) was significantly lower than that of patients with low or no NEK6 expression (blue line).

In addition, Pearson correlation analysis revealed significant differences between high NEK6 expression and clinicopathological factors, including histological grade (*P*=0.008) and metastasis (*P*=0.006), in OC patients. A total of 110 patients with OC were subjected to survival analysis via Kaplan‒Meier survival curves, which revealed that OC patients with NEK6 overexpression had a poorer overall survival rate than did those with low NEK6 expression (Fig. 3) (*P*=0.025).

These results indicate that NEK6 is highly expressed in the tumor tissues of OC patients and that its high expression is associated with the progression of OC, which may be a poor factor for OC.

### NEK6 overexpression in OC tissues and cells

**Figure 4:**
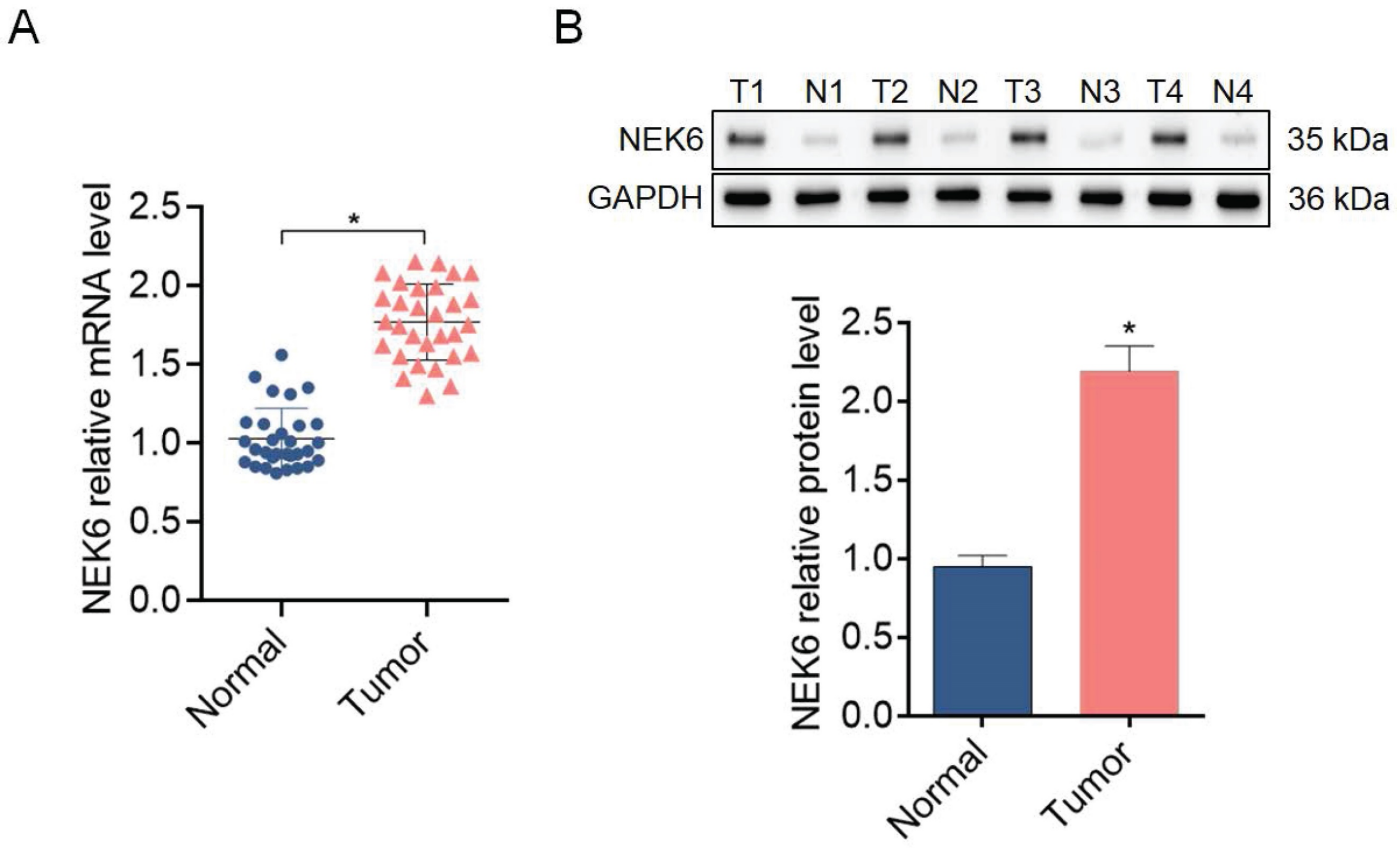
The expression levels of NEK6 in tissues from OC patients. **A.** NEK6 mRNA levels in OC tissues (n=30) compared with those in normal ovary tissues (n=30) were detected via RT‒qPCR. **B.** NEK6 protein levels in OC tissues (n=30) compared with those in normal ovary tissues (n=30) were detected by Western blotting. T, tumor tissues; N, normal ovarian tissues. *P<0.05, compared with normal ovary tissues.

**Figure 5:**
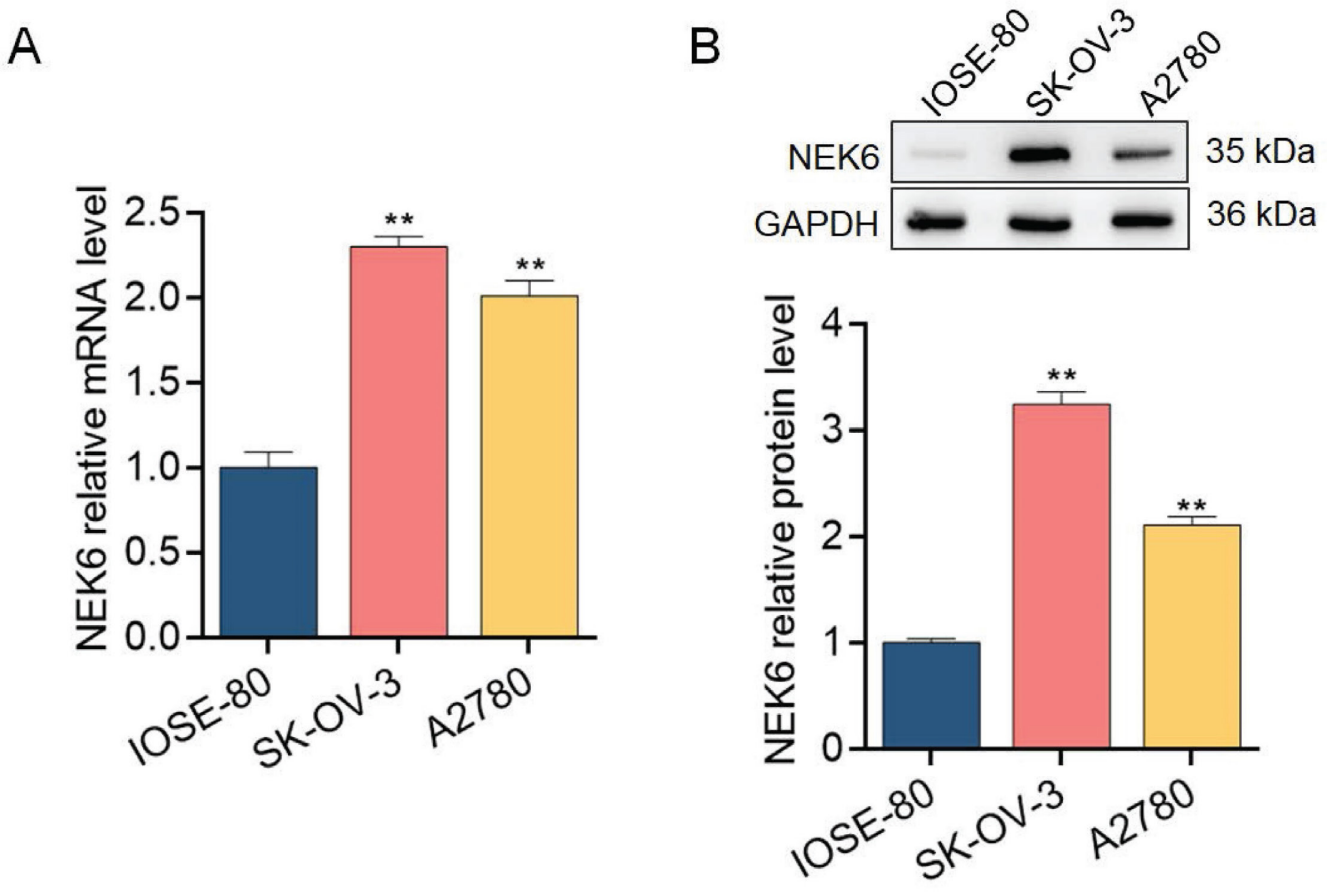
Expression levels of NEK6 in OC cells. **A.** NEK6 mRNA levels in the OC cell lines SK-OV-3 and A2780 were detected by RT‒qPCR. **B.** NEK6 protein levels in SK-OV-3 and A2780 cells detected by Western blotting. **P<0.01, compared with the normal ovary epithelial cell line IOSE-80.

To further explore the expression profile of NEK6 in OC tissues and cells, 30 samples of fresh OC tissues and paired normal ovarian tissues and the OC cell lines SK-OV-3 and A2780 were used for detection via RT‒qPCR and Western blotting. NEK6 mRNA and protein levels were greater in OC tissues than in normal tissues (*P*<0.05) (Fig. 4), and NEK6 mRNA and protein levels were also greater in SK-OV-3 and A2780 cells than in the normal ovary epithelial cell line IOSE-80 (*P*<0.05) (Fig. 5).

These results indicate that both NEK6 mRNA and protein are highly expressed in OC tumor tissues and cell lines, including SK-OV-3 and A2780 cells.

### NEK6 knockdown with siRNAs in OC cells

To further analyze the functions of NEK6 in OC cells, predesigned siRNAs (si-NEK6-1, si-NEK6-2 and si-NEK6-3) were used to knock down endogenous NEK6 expression.

**Figure 6:**
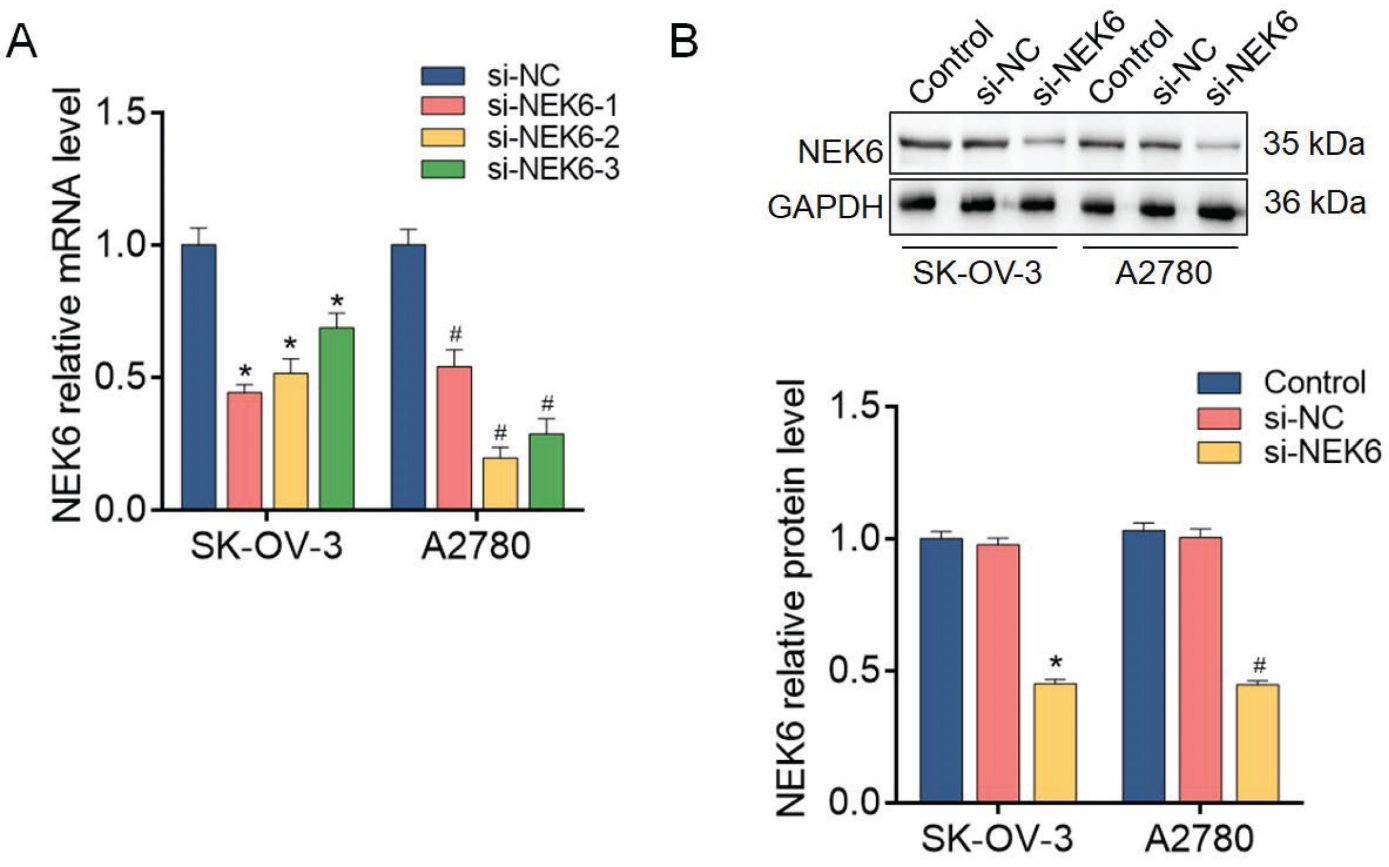
NEK6 is inhibited by siRNAs in OC cells. **A.** The mRNA levels of NEK6 were inhibited by predesigned siRNAs (si-NEK6-1, si-NEK6-2, and si-NEK6-3), as detected by RT‒qPCR. si-NEK6-2 was selected for subsequent assays and named si-NEK6. **B.** The protein levels of NEK6 were inhibited by predesigned siRNAs, as detected by Western blotting. *P<0.05, compared with si-NC-treated SK-OV-3 cells; ^#^*P*<0.05, compared with si-NC-treated A2780 cells.

NEK6 mRNA levels were significantly reduced by si-NEK6-1, si-NEK6-2 and si-NEK6-3, both in SK-OV-3 and A2780 cells, compared with those in si-NC-treated cells (*P*<0.05) (Fig. 6). The knockdown efficiency of si-NEK6-2 was apparent in both cell lines; thus, it was used for the following functional assays and named si-NEK6. In addition, the protein levels of NEK6 were significantly inhibited by si-NEK6 in SK-OV-3 and A2780 cells (Fig. 6).

These results indicate that the expression of NEK6 in OC cells can be effectively knocked down by specific siRNAs.

### Inhibitory effects of NEK6 knockdown on OC cell growth, migration, invasion and apoptosis

**Figure 7:**
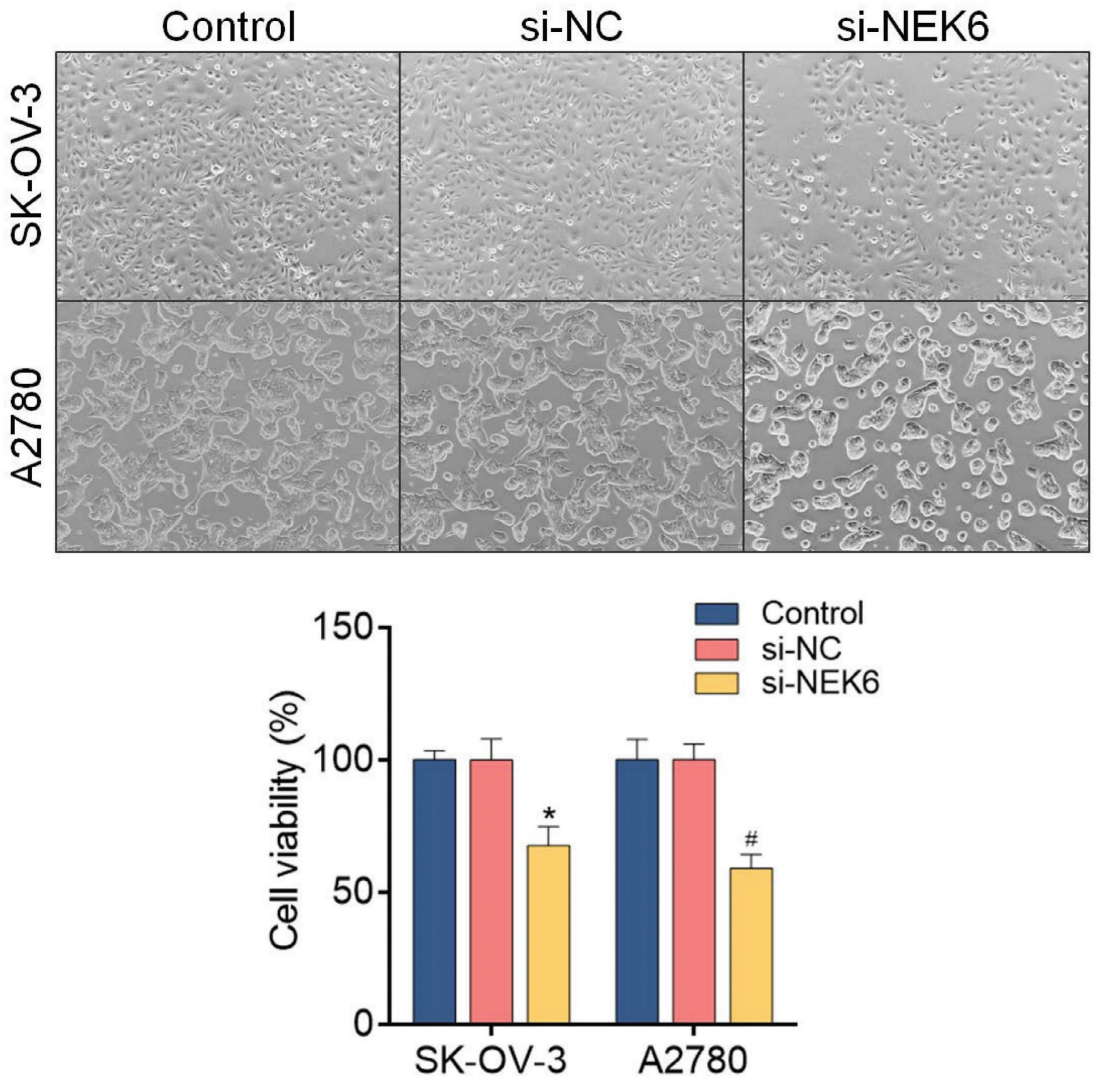
Effect of siRNA-mediated NEK6 inhibition on the growth of SK-OV-3 and A2780 cells detected via the MTT assay. *P<0.05, compared with si-NC-treated SK-OV-3 cells; ^#^*P*<0.05, compared with si-NC-treated A2780 cells.

To determine the inhibitory effects of NEK6 knockdown on OC cell growth, an MTT assay was used. The results revealed that, compared with si-NC, si-NEK6 inhibited the growth of SK-OV-3 and A2780 cells at 48 h and 72 h (*P*<0.05) (Fig. 7).

**Figure 8:**
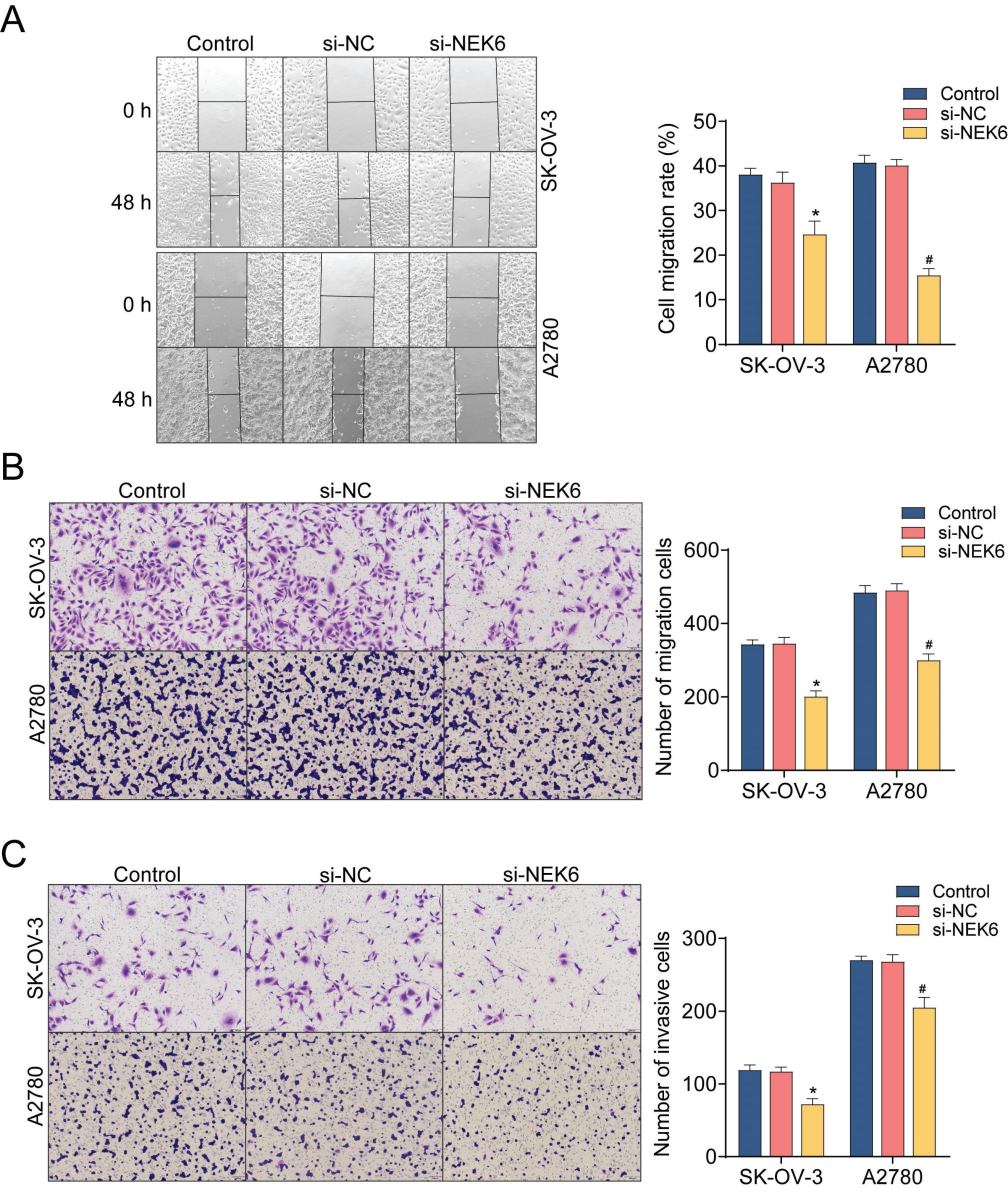
Effects of NEK6 knockdown on the migration, invasion and apoptosis of OC cells. **A.** Migration abilities of OC cells detected by the wound-healing assay. **B.** The migration abilities of OC cells detected by the transwell assay. **C.** The invasion abilities of OC cells detected by the Matrigel-based transwell assay. **D.** The apoptosis of OC cells was detected by Annexin V-FITC/PI staining and FCM. *P<0.05, compared with si-NC-treated SK-OV-3 cells; ^#^*P*<0.05, compared with si-NC-treated A2780 cells.

Both a wound healing assay and a transwell assay were used to observe the inhibitory effect of NEK6 knockdown on OC cell migration. The results revealed that, compared with si-NC, si-NEK6 significantly inhibited the migration of SK-OV-3 and A2780 cells (*P*<0.05) (Fig. 8A & B). Additionally, the invasive abilities of OC cells were detected by a Matrigel-based assay, which revealed that, compared with si-NC, si-NEK6 significantly inhibited the invasive abilities of SK-OV-3 and A2780 cells (*P*<0.05) (Fig. 8C).

To evaluate the effect of NEK6 knockdown on OC apoptosis, flow cytometry with Annexin-V-FITC/PI double staining was used. After 48 h of treatment with si-NEK6, the apoptosis of SK-OV-3 and A2780 cells was clearly promoted compared with that of si-NC-treated cells (*P*<0.05) (Fig. 8D).

These results indicate that NEK6 knockdown can inhibit OC cell growth, migration and invasion but can inhibit cell apoptosis.

## Discussion

The global prevalence of OC is increasing, seriously affecting women's health. Epithelial cancer is the most common type of OC, but ovarian epithelial cancer accounts for the highest mortality rate among various gynecological tumors, posing a serious threat to women's lives 21. Owing to the complex embryonic development, tissue anatomy, and endocrine function of the ovary, early symptoms are not typical, and distinguishing the tissue type and benign and malignant ovarian tumors before surgery is quite difficult. Epithelial ovarian cancer is prone to metastasis and invasion, and only 30% of patients present with tumors limited to the ovaries during surgery. Therefore, early diagnosis poses a major challenge 22.

The main characteristic of cancer is abnormal cell proliferation, which may be caused by an imbalance in cell cycle regulation 23. Studies have shown that the expression of NEKs in the G1 phase of the cell cycle is low but increases in the S and G2 phases; however, when cells enter mitosis, their expression decreases 24. NEK activity peaks in the S and G2 phases and leads to the separation of centrosomes in the G2/M phase 25. Therefore, high expression of NEKs leads to chromosomal instability and aneuploidy in cancer cells. In addition, high expression of NEKs activates several carcinogenic pathways, leading normal cells to transform into cancer cells through excessive proliferation and metastasis 26. Therefore, recent studies have focused on the correlation between NEKs and various cancers, revealing their involvement in gastric cancer 27,28, prostate cancer 5, colon cancer 29, breast cancer 30,31, hepatocellular carcinoma 32, glioblastoma 33,34, pancreatic cancer 35 and cervical cancer 36.

As an important member, NEK6 has also been found to be overexpressed in various cancers. NEK6 has been reported to be a central kinase responsible for the development of castrated tolerant prostate cancer 5, and knocking out NEK6 can reduce clonogenesis, proliferation, cell viability and mitochondrial activity and further increase intracellular reactive oxygen species (ROS) levels. In head and neck squamous cell carcinoma (HNSCC), NEK6 is significantly upregulated, indicating poor prognosis in HNSCC patients. An increase in NEK6 expression leads to immune cell infiltration and an increase in various immune checkpoints 11. In hepatocellular carcinoma, NEK6 is highly expressed and promotes cell proliferation 12. NEK6 is significantly upregulated in clear cell renal cell carcinoma (CCRCC) and significantly promotes CCRCC cell proliferation, migration, and invasion and inhibits apoptosis 37. NEK6 is overexpressed in breast cancer, and its high expression is associated with histological grade, tumor size and TNM stage. Cox regression analysis revealed that NEK6 expression is an independent prognostic factor for cancer. NEK6 plays a role in promoting the proliferation of breast cancer cells and may become a promising therapeutic target for breast cancer 13.

In this study, we found that NEK6 was highly expressed in most human cancers, including OC, via a pancancer analysis with bioinformatics tools. Furthermore, among a total of 110 OC patients, IHC revealed that NEK6 was positively stained in tumor tissues and negatively stained in normal tissues and that its overexpression was correlated with clinicopathological factors, such as histological grade and metastasis. In addition, OC patients with high NEK6 expression had a lower survival rate. Moreover, when NEK6 was knocked down with siRNAs, the growth, migration and invasion of OC cells were inhibited, while apoptosis was significantly promoted.

In summary, NEK6 is an oncogene in OC and may be a poor prognostic factor, suggesting that NEK6 may be a novel candidate therapeutic target for OC and that inhibiting NEK6 may be an effective strategy for OC treatment.

## Figures and Tables

**Table 1:** siRNA sequences used to knock down NEK6 expression.

Name	Sequence (5'-3')	
si-NEK6_1	Sense	CAGAUGAUCAAGUACUUUAdTdT
	Antisense	UAAAGUACUUGAUCAUCUGdTdT
si-NEK6_2	Sense	CGGAGAGGACAGUAUGGAAdTdT
	Antisense	UUCCAUACUGUCCUCUCCGdTdT
si-NEK6_3	Sense	CUCCGAGAAGUUACGAGAAdTdT
	Antisense	UUCUCGUAACUUCUCGGAGdTdT
si-NC	Sense	UUCUCCGAACGUGUCACGUdTdT
	Antisense	ACGUGACACGUUCGGAGAAdTdT

**Table 2:** Correlations between NEK6 expression and clinicopathological factors in OC patients.

Clinicopathological parameters	No. cases	NEK6 expression, n		*Pearson x^2^*	*P*
		-/+	++/+++		
Age				0.056	0.826
≤50	32	11	21		
>50	78	25	53		
Pathological type				1.209	0.877
Serous carcinoma	76	24	52		
Mucinous carcinoma	5	1	4		
Endometrioid carcinoma	12	5	7		
Clear cell carcinoma	10	3	7		
Others	7	3	4		
FIGO stage				0.693	0.423
I/II	61	22	39		
III/Ⅳ	49	14	35		
Histological grade				7.259	0.008*
I/II	76	31	45		
III	34	5	29		
Metastasis				7.628	0.006*
No	87	34	53		
Yes	23	2	21		

* represents statistical significance, *P*<0.05
